# The dual role of SOX17 in the occurrence and development of early colorectal cancer

**DOI:** 10.1186/s43556-024-00201-2

**Published:** 2024-09-16

**Authors:** Xinming Su, Jianqiao Shentu, Ruixiu Chen, Shiwei Duan

**Affiliations:** Department of Clinical Medicine, Hangzhou City University, Hangzhou, Zhejiang China

**Keywords:** SOX17, Colorectal cancer, Immune evasion, Tumor initiation, Therapeutic targets

## Main text

Tumor immune evasion, characterized by the adept evasion of detection and assault by the body's immune system, represents a crucial mechanism facilitating the survival and proliferation of cancer cells within the body [1]. This phenomenon is widely recognized as a significant driver behind the onset and progression of numerous malignancies. Colorectal cancer (CRC), a prototypical malignancy renowned for its robust immunosuppressive microenvironment, frequently elicits immune evasion due to the accumulation of genetic mutations. Nevertheless, our comprehension of the specific mechanisms governing this immune evasion behavior in precancerous lesions or early-stage cancers remains relatively limited.

**Fig. 1:**
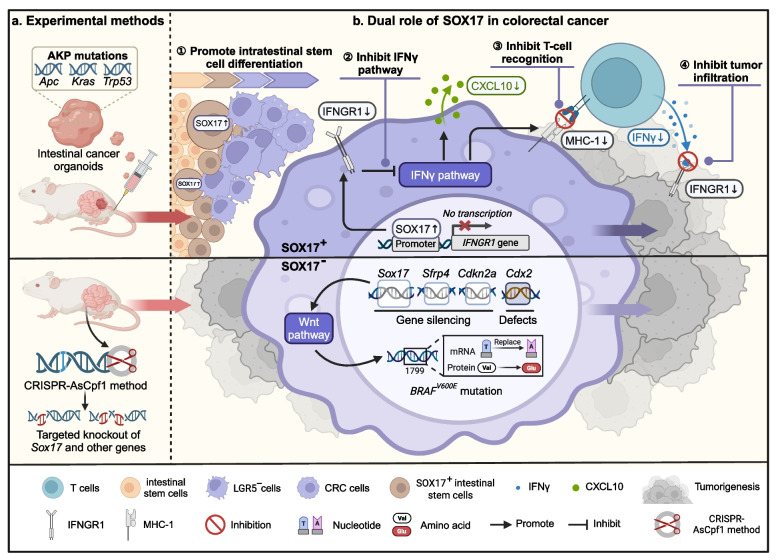
The Dual Role of SOX17 in CRC Initiation and Progression. A recent study has shed light on the impact of SOX17 expression in the context of intestinal cancer organoids harboring *Apc*, *Kras*, and *Trp53* mutations (termed AKP). Upon implantation into mice and subsequent induction of malignant transformation, the study unveiled a significant influence of SOX17. Specifically, SOX17 was found to attenuate the IFNγ signaling pathway within early CRC cells and precancerous cells, consequently diminishing the production of MHC-I protein and CXCL10. This inhibition rendered T cells ineffective in executing their cytotoxic function. Moreover, SOX17 demonstrated the capacity to drive the differentiation of intestinal stem cells into LGR5^−^ intestinal stem cells characterized by low MHC-I expression. This phenomenon enabled these cells to evade immune surveillance, thereby fostering the initiation and progression of CRC. Contrary to these findings, another study presents an alternative perspective. Employing CRISPR-AsCpf1 technology, the study conducted simultaneous or combined targeted knockout of four pivotal genes, including *Sox17*, in mouse colon organoids. The outcomes revealed that the deletion of *Sox17*, along with two additional genes (*Sfrp4* and *Cdkn2a*), in CRC context could synergize with the loss of *Cdx2*. This cooperative effect further propelled CRC progression instigated by the *BRAF*^*V600E*^ mutation in experimental stem cells through the aberrant activation of Wnt signaling. This discovery unveils a novel dimension in our comprehension of the intricate mechanisms governing CRC. Abbreviations: CRC, colorectal cancer

SOX17, initially identified as a transcription factor associated with endoderm and fetal foregut development, has recently emerged as a significant player in cancer progression by orchestrating cellular reprogramming across various cancer types. Notably, recent publications in *Nature *[2] and *Nature Communications *[3] have unveiled the pivotal role of SOX17 in driving the transformation of intestinal stem cells into tumors. However, it is noteworthy that the conclusions drawn by these two research groups regarding the oncogenic role of SOX17 in early CRC diverge significantly (Fig. 1).

Ömer H. Yilmaz and colleagues elucidated the pivotal role of SOX17 in shielding precancerous cells from immune system assault. Using CRISPR-Cas9 editing and Cre-mediated recombination, they engineered and implanted mouse colon cancer tissues with *Apc*, *Kras*, and *Trp53* mutations (AKP) into mice. Advanced RNA-seq and ATAC-seq techniques revealed that SOX17 was identified as a significantly upregulated factor in CRC, playing a critical role in transcriptional regulation by modulating chromatin accessibility. CRISPR-Cas9 was then used to knock out *Sox17*, revealing that SOX17 promotes an immunosuppressive environment and tumor progression by hindering immune clearance mechanisms. SOX17 exhibits the capability to directly bind to the promoter region of IFNGR1, a component of the interferon-gamma (IFNγ) receptor. This binding event results in the suppression of IFNGR1 expression, effectively impeding the IFNγ signaling cascade within CRC cells and precancerous adenoma cells. As a result, levels of MHC-I and CXCL10 are diminished, thereby hindering the mechanism of CD^8+^ T cell-mediated tumor elimination. These intricate biological processes underscore the SOX17's pivotal role in modulating tumor immune responses. Additionally, SOX17 facilitated the differentiation of intestinal stem cells into counterparts with diminished MHC-I expression, thus enabling immune evasion in early adenomas and CRC. Notably, these observations were corroborated in organoids derived from human intestinal stem cells, underscoring the evolutionary conservation of SOX17's regulatory impact on antitumor immunity and highlighting its potential as a biomarker and therapeutic target.

Conversely, the research findings published by Hariharan Easwaran's team in *Nature Communications* present a contrasting narrative. Utilizing CRISPR-AsCpf1 technology, researchers precisely targeted four crucial genes within mouse colon organoids for simultaneous or combined knockout. Among these genes, *Sox17* stands out, frequently experiencing silencing due to promoter hypermethylation in CRC. The investigation successfully revealed that deleting *Sox17*, along with two other equally significant genes—*Sfrp4*, which encodes a Wnt repressor, and *Cdkn2a*, which induces cell cycle arrest—operates in synergy with the deletion of *Cdx2*, crucial to the transcriptional program of intestinal development. Notably, the proximal colon-specific *BRAF*^*V600E*^ mutation-driven tumorigenesis is bolstered by the activation of the Wnt signaling pathway. This seminal finding unveils fresh perspectives on CRC pathogenesis, offering valuable insights for prospective therapeutic interventions. This suggests a potential inhibitory role for SOX17 in CRC.

Indeed, SOX17 has previously been recognized as a negative regulator of the canonical Wnt signaling pathway and has demonstrated antitumor properties in certain cancer contexts [4, 5]. The disparate outcomes reported by these two teams underscore the potential dual role of SOX17, akin to the extensively studied TP53, wherein it may function as both a tumor suppressor and a promoter of tumor progression under distinct circumstances. These discrepancies may arise from variations in experimental conditions, cell types and states, as well as potential isoform-specific functions of SOX17 and other influencing factors.

The CRISPR-AsCpf1 technology used by Hariharan Easwaran's team, compared to the CRISPR-Cas9 editing used by Ömer H. Yilmaz's team, has terminal structure limitations, leading to complex and uncertain editing results. Easwaran's team focused on female mice due to the influence of X chromosome genes and sex hormones on cell growth, division, and gene expression, which significantly impacted editing efficiency and outcomes. Additionally, although both studies utilized CRISPR lentiviral vectors for mouse organoid editing, the differences in guide RNA sequences contributed to varying results. Given the substantial interindividual differences, it is imperative to explicitly address these nuances in distinct cell types and states explicitly. Furthermore, post-transcriptional and post-translational regulatory mechanisms under diverse conditions may induce fluctuations in mRNA and protein expression levels, presenting another potential explanation for conflicting experimental outcomes. Hence, meticulous exploration and validation of these intricate factors are imperative for accurately elucidating the function of SOX17 and its underlying mechanisms across varying conditions. Moreover, despite both studies using CRISPR lentiviral vectors for mouse organoid editing, discrepancies arise from the utilization of distinct guide RNA sequences. Such inconsistencies may induce variations in on-target and off-target activities, hinting at the possibility of SOX17 harboring specific transcript isoforms with divergent functional attributes. Concurrently, the diverse modifications and states induced by various triggering factors, alongside disparities in their impact on downstream pathways under differing contexts, may yield diametrically opposed experimental results.

Notwithstanding the controversy, the pioneering research conducted by these two teams engenders fresh perspectives for subsequent investigations. The diminishing degree of SOX17 expression in CRC progression endows it with a unique potential as an early diagnostic biomarker for CRC. However, as transcription factors like SOX17 are typically challenging to target directly with drugs, therapeutic interventions may necessitate the design of targeted drugs aimed at their regulatory networks. While the findings of these studies unveil the intricate regulatory network associated with SOX17 in CRC, further exploration and validation are imperative to fill existing knowledge gaps.

In summary, both studies highlight the crucial role of SOX17 in the early development of CRC, particularly in the formation of precancerous lesions and the evasion of immune surveillance by cancer cells. These findings provide valuable insights for developing new detection and treatment strategies for early-stage CRC. SOX17 likely plays a dual role similar to TP53 in various cancers, but this has not been thoroughly investigated. As research advances, SOX17 may emerge as a key biomarker for the early diagnosis of CRC and an important target for future immunotherapy. Further studies could reveal more mechanisms of SOX17's action in CRC and other cancers, potentially leading to breakthroughs in early cancer diagnosis and treatment, ultimately providing patients with more effective therapeutic options.

## Data Availability

Not applicable.
